# The anti-malarial atovaquone increases radiosensitivity by alleviating tumour hypoxia

**DOI:** 10.1038/ncomms12308

**Published:** 2016-07-25

**Authors:** Thomas M. Ashton, Emmanouil Fokas, Leoni A. Kunz-Schughart, Lisa K. Folkes, Selvakumar Anbalagan, Melanie Huether, Catherine J. Kelly, Giacomo Pirovano, Francesca M. Buffa, Ester M. Hammond, Michael Stratford, Ruth J. Muschel, Geoff S. Higgins, William Gillies McKenna

**Affiliations:** 1 CRUK/MRC Oxford Institute for Radiation Oncology, Old Road Campus Research Building, Roosevelt Drive, Oxford OX3 7DQ, UK; 2 OncoRay – National Center for Radiation Research in Oncology, Faculty of Medicine and University Hospital Carl Gustav Carus, TU Dresden, and Helmholtz-Zentrum Dresden–Rossendorf, Institute of Radiooncology, Dresden, P.O. Box 41, 01307, Germany

## Abstract

Tumour hypoxia renders cancer cells resistant to cancer therapy, resulting in markedly worse clinical outcomes. To find clinical candidate compounds that reduce hypoxia in tumours, we conduct a high-throughput screen for oxygen consumption rate (OCR) reduction and identify a number of drugs with this property. For this study we focus on the anti-malarial, atovaquone. Atovaquone rapidly decreases the OCR by more than 80% in a wide range of cancer cell lines at pharmacological concentrations. In addition, atovaquone eradicates hypoxia in FaDu, HCT116 and H1299 spheroids. Similarly, it reduces hypoxia in FaDu and HCT116 xenografts in nude mice, and causes a significant tumour growth delay when combined with radiation. Atovaquone is a ubiquinone analogue, and decreases the OCR by inhibiting mitochondrial complex III. We are now undertaking clinical studies to assess whether atovaquone reduces tumour hypoxia in patients, thereby increasing the efficacy of radiotherapy.

## Abstract (web summary)

Tumour hypoxia reduces the efficacy of radiotherapy. Starting from a drug screen, here the authors demonstrate that the anti-malarial, atovaquone, reduces the oxygen consumption rate of cancer cells by inhibition of mitochondrial complex III and sensitises to radiotherapy by reducing tumour hypoxia.

## Graphical abstract

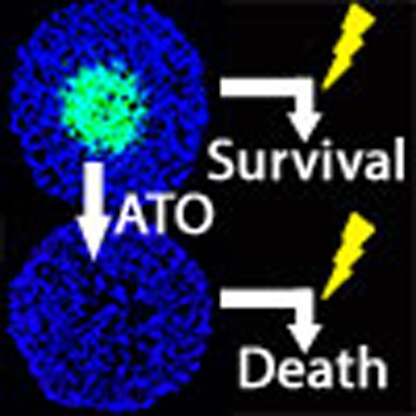

Solid malignancies frequently have regions of hypoxia^1^. Many studies have focused on the abnormal tumour vasculature as a determinant of tumour hypoxia. However, tumour oxygenation reflects the balance between delivery and consumption, and because tumour cells have a high level of oxygen consumption, and reduced delivery of oxygen to the tumour due to abnormal vasculature, both components play a role^2^. Hypoxia is associated with poor clinical outcomes due to both local recurrence and an increased probability of metastasis^3^. Gray and his colleagues in the 1950s were the first to suggest that hypoxia would affect the outcome of radiotherapy because hypoxic tumour cells are up to three times more radioresistant than normoxic tumour cells^1^,^3^,^4^. This radioresistance is due to the absence of the oxygen enhancement effect, which is a result of the direct physicochemical reaction of oxygen with the broken ends of the DNA strands that result from radiation, creating stable organic peroxides that are more difficult for the cell to repair.

Vascular remodelling has been used previously as a strategy to relieve tumour hypoxia and improve radiation response, and it has been demonstrated that a number of signal transduction inhibitors have such an effect^5^. A possible alternative strategy to reduce tumour hypoxia, and therefore to increase radiosensitivity, is to reduce the cellular oxygen consumption rate (OCR). Reduction of the OCR in three-dimensional (3D) multicellular tumour spheroids causes a decrease in the volume of the central region of hypoxia by increasing the availability of oxygen throughout the spheroid^6^,^7^,^8^. These studies in spheroids demonstrate that reducing the OCR can alleviate hypoxia in an avascular system. Mathematical modelling suggests that a 30% decrease in the OCR would even abolish severe tumour hypoxia, and that this could be a more effective approach to reduce hypoxia than attempts at elevating blood flow or increasing the oxygen levels in blood^8^. An additional advantage of assessing the OCR is that it is amenable to high-throughput screening, which vascular remodelling is not. Drugs that reduce the OCR have been identified previously by screens designed to identify inhibitors of the HIF-1 pathway^9^,^10^, but to our knowledge, no high-throughput screens designed to identify novel inhibitors of the OCR in cancer cells have been published.

The anti-diabetic, metformin, is the only FDA-approved drug that has been shown to reduce both the OCR and tumour hypoxia, and its mechanism of action is inhibition of mitochondrial complex I (refs 11, 12). However, at pharmacological concentrations of metformin there is a reduction in the OCR of only 10–20%, suggesting that compounds with a more significant effect on the OCR may have a more profound effect on tumour hypoxia^12^. Thus there is a need to identify more drugs that alleviate tumour hypoxia by reducing the OCR.

To identify drugs that decrease the OCR of cancer cells and could be used as modifiers of tumour hypoxia in a clinical setting, we screen a library of 1,697 FDA-approved compounds. This screen identifies atovaquone, a drug primarily used to treat malaria and pneumocystis pneumonia. Atovaquone reduces the OCR by inhibition of mitochondrial complex III (the cytochrome *bc*_1_ complex) at pharmacologically achievable concentrations, alleviates hypoxia both in spheroids and in xenografted tumours, and causes a significant tumour growth delay in combination with radiation.

## Results

### High-throughput screening to assess oxygen consumption

**Figure 1:**
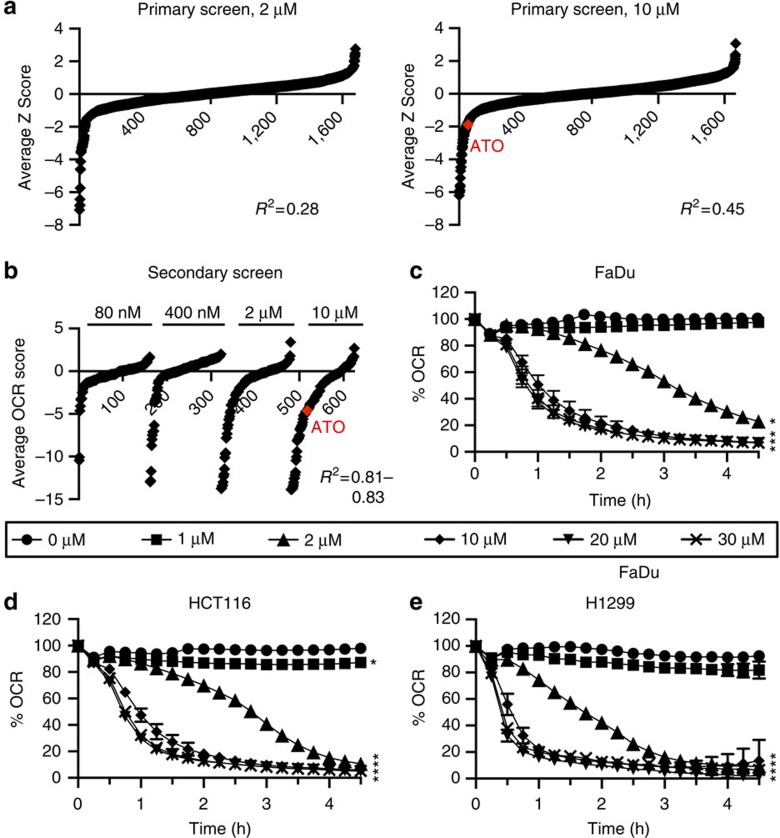
A screen to discover compounds that decrease the OCR of FaDu cells. (**a**) For the primary screen, FaDu cells were grown in galactose-containing medium and incubated with a library of 1697 FDA-approved compounds for 24 h. The mitochondrial specific OCR was measured, and subsequent hoechst staining was used to correct for cell number. (**b**) For the secondary screen, FaDu cells were grown in glucose-containing medium, incubated with the compounds for 24 h, and then the mitochondrial-specific OCR was measured and normalized to cell number. (**c**–**e**) The OCR of FaDu, HCT116 and H1299 cells were measured for 4.5 h after injection of atovaquone. The % OCR post injection is shown relative to the DMSO control and normalized to the relative cell number obtained by hoechst staining at the end of the experiment. The data are representative of three independent experiments (mean±s.d.). One-way ANOVA were performed to assess statistical significance at 4.5 h after injection of atovaquone with Bonferroni post correction (*P<0.0001). ATO, atovaquone.

To identify compounds that reduce the OCR, FaDu hypopharyngeal carcinoma cells were incubated with 1,697 FDA-approved compounds at 2 or 10 μM for 24 h (Fig. 1a). The growth medium was replaced with an assay medium containing 5 mM galactose, 5 mM pyruvate and 4 mM glutamine. Growth in galactose promotes oxidative phosphorylation^13^. The mitochondrial-specific OCR was determined by measuring basal OCR and subtracting the OCR measured following injection of 2 μM antimycin A to inhibit mitochondrial respiration. The OCR measurements were corrected for cell number using the relative Hoechst fluorescence of the cells fixed immediately after the assay. Compounds that caused a reduction in cell number of more than 66% compared with the DMSO control wells for each plate were excluded. The compounds with the top 130 rank products were chosen for the secondary screen (Supplementary Data 1). The secondary screen was conducted using the same protocol as the primary screen, except FaDu cells were incubated with the compounds at 80 nM, 400 nM, 2 μM or 10 μM, and the assay medium contained 5 mM glucose instead of 5 mM galactose to more closely mimic the physiological setting (Fig. 1b and Supplementary Data 1). The highest ranked compounds that caused a decrease in the OCR in FaDu cells at 10 μM and are approved for systemic use in humans are shown in Table 1.

Compounds were subsequently excluded from further study based on a range of criteria (Supplementary Table 1): Known *in vivo* hypoxia modifiers or radiosensitisers such as acriflavinium^14^, compounds with an unsuitable safety profile such as emetine^15^, anti-helminths with poor bioavailability such as pyrvinium^16^, and compounds for which the maximum achievable plasma concentration in patients is lower than the concentration required to cause a significant reduction in the OCR, such as dactinomycin^17^. The anti-malarial, atovaquone was selected for further evaluation, because it is not known to reduce hypoxia *in vivo*, it has an excellent safety profile, it has ideal pharmacokinetic properties for a hypoxia modifier, and has low cost^18^.

To evaluate the effect of atovaquone on other cell lines, the OCR was measured for 4.5 h after its addition. A significant decrease in the OCR of 78.7% to 90.0% compared with the DMSO control was observed after 2 h at 10 μM in FaDu, HCT116 colorectal carcinoma, DLD-1 colorectal adenocarcinoma, H1299 lung carcinoma, A549 lung carcinoma, H460 large cell lung carcinoma, MCF7 breast ductal carcinoma and T24 bladder carcinoma cells, with a lesser but still significant decrease of 57.6% in PSN-1 pancreatic adenocarcinoma cells (Fig. 1c–e and Supplementary Fig. 1a). This was a dose-dependent effect, with a smaller, but still significant, decrease observed at 2 μM, and no significant decrease observed at 1 μM. There was no significant effect on the cell number relative to the DMSO control in FaDu, HCT116 and H1299 cells at concentrations between 10 and 100 μM, or in the other cell lines at 10 μM during the course of the experiment (Supplementary Fig. 1b,c). Statistically significant decreases in cell survival of 25.9–56.2% were observed in FaDu, HCT116 and H1299 cells after a longer incubation of 24 h with 20 or 30 μM atovaquone, but cell survival was partially rescued following 4 days recovery in drug-free medium (Supplementary Fig. 1d). The widely used anti-cancer therapeutics, 5-fluorouracil, docetaxel and cytarabine caused a significant decrease in FaDu cell survival of 18.7–37.8% after 24 h incubation at either 2 μM or 10, but this did not correspond with a significant decrease in the OCR (Supplementary Fig. 2a,b). This demonstrates that a decrease in cell survival observed following protracted incubation with an anti-proliferative drug does not necessarily correlate with a decrease in the OCR. Thus atovaquone reduces the OCR in cancer cells from a wide range of tumour types.

### ATO affects spheroid hypoxia and radiation response

**Figure 2:**
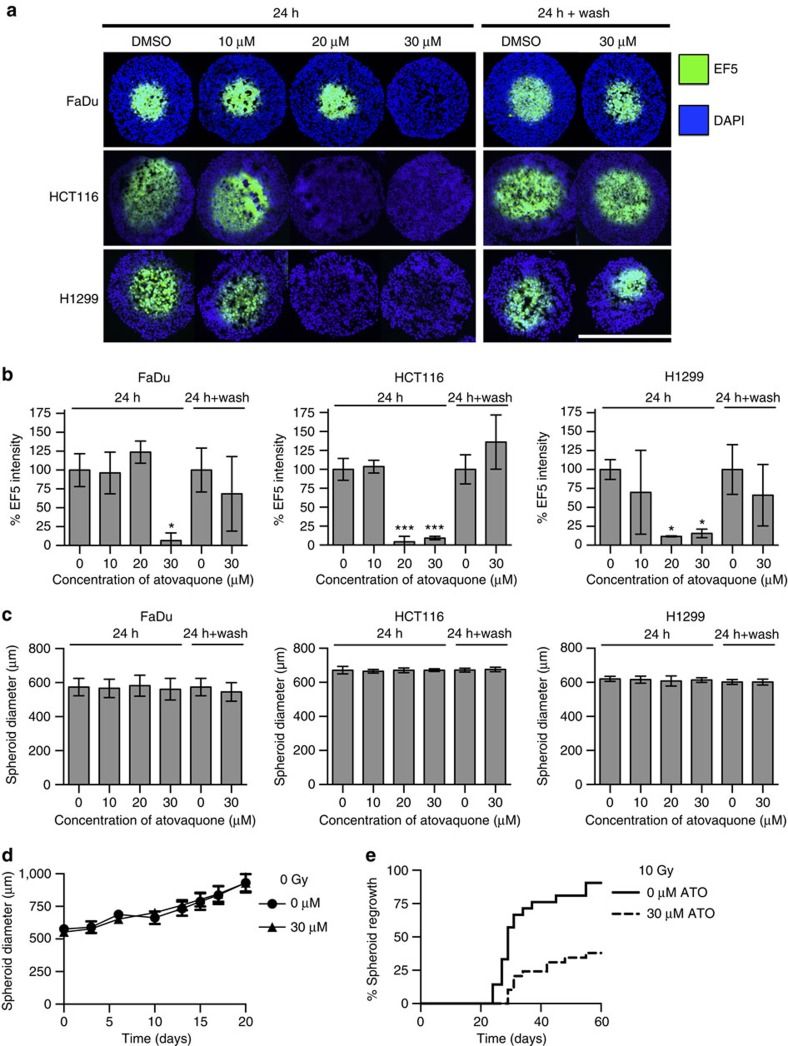
Atovaquone alleviates spheroid hypoxia and improves radiation response. (**a**,**b**) FaDu, HCT116 and H1299 spheroids were treated with DMSO or atovaquone either for 24 h, or for 24 h followed by recovery for 24 h in drug-free medium, as indicated. Hypoxia was assessed by staining central spheroid sections for EF5 (green), with DAPI as a nuclear counterstain (blue). Scale bar, 600 μm. % Mean EF5 fluorescence intensity is presented relative to the DMSO controls. (**c**) Average spheroid diameter. Hypoxia was assessed in at least five spheroids per treatment for each experiment (*n*=3). One-way ANOVA was performed to assess statistical significance with Bonferroni post correction (*P>0.05, ***P<0.001). (**d**,**e**) FaDu spheroids were treated with DMSO or 30 μM atovaquone for 24 h, and then received either 0 Gy (**d**) or 10 Gy (**e**). After treatment, the spheroids were allowed to recover in drug-free medium. Time post treatment is shown (days). One replicate of the experiment was conducted with at least 22 spheroids in each treatment group. All values are presented as mean±s.d.

Reduction of the OCR in 3D multicellular tumour spheroids can lead to a decrease in hypoxia^6^,^7^,^8^. FaDu, HCT116 and H1299 spheroids of 550–650 μm in diameter were treated with 10, 20 or 30 μM atovaquone for 24 h, and the hypoxic fraction was evaluated by EF5 staining. There was complete loss of hypoxia in FaDu spheroids at 30 μM, and in HCT116 and H1299 spheroids at 20 μM atovaquone (Fig. 2a,b). It is possible that a decrease in EF5 staining could be caused by an increase in cell death^7^. To exclude this possibility, the spheroids were washed and allowed to recover in drug-free medium for 24 h, resulting in the return of hypoxia (Fig. 2a,b). None of the treatments caused a significant decrease in the diameter of FaDu, HCT116 or H1299 spheroids (Fig. 2c). Taken together, these results indicate that the observed decrease in the OCR induced by atovaquone corresponds to a decrease in hypoxia in an avascular 3D tumour environment.

To determine whether the alleviation of spheroid hypoxia translated to an improvement in radiation response, FaDu spheroids were treated for 24 h with 30 μM atovaquone, irradiated at 10 Gy, and then allowed to recover in fresh medium. For unirradiated spheroids, atovaquone treatment did not cause a significant difference in spheroid growth after 20 days (Fig. 2d). Irradiation at 10 Gy caused dissociation of both the DMSO and atovaquone-treated spheroids, but 90.91% of the DMSO-treated spheroids reformed by 60 days after irradiation, compared with only 37.93% of atovaquone treated spheroids (Fig. 2e). This decrease in irradiated spheroid regrowth following atovaquone treatment is likely due to the observed reduction in spheroid hypoxia.

### ATO inhibits mitochondrial complex III

**Figure 3:**
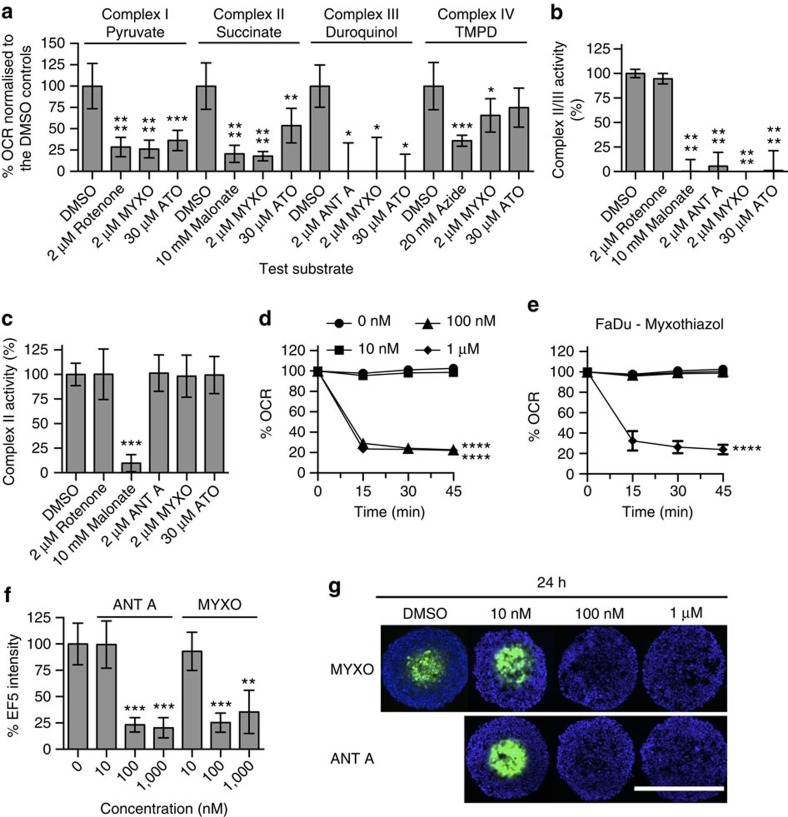
Atovaquone inhibits complex III activity. (**a**) The effect of atovaquone on complex I-, II-, III- or IV-dependent respiration in FaDu cells permeabilized with 0.005% digitonin. The % OCR was measured immediately after permeabilization. (**b**) Complex II/III activity was measured in mitochondria isolated from FaDu cells 15 min after compound addition. (**c**) Complex II activity was measured in mitochondria isolated from FaDu cells 15 min after compound addition. (**d**,**e**) The OCR of FaDu cells was measured for 45 min after injection of antimycin A (**d**) or myxothiazol (**e**). The % OCR post injection is shown relative to the DMSO control and normalized to the relative cell number obtained by hoechst staining at the end of the experiment. The data is representative of three independent experiments. (**f**,**g**) FaDu spheroids were treated with DMSO, antimycin A or myxothiazol for 24 h. Hypoxia was assessed by staining central spheroid sections for EF5 (green), with DAPI as a nuclear counterstain (blue). Scale bar, 600 μm. % Mean EF5 fluorescence intensity is presented relative to the DMSO controls. Hypoxia was assessed in at least five spheroids per treatment for each experiment (*n*=3). One-way ANOVA with Bonferroni was performed for all of the experiments (*n*=3, mean±s.d., ****P<0.0001, ***P<0.001, **P<0.01, *P<0.05). ANT A, antimycin A; ATO, atovaquone; MYXO, myxothiazol.

As a ubiquinone analogue, the primary clinical mechanism of action of atovaquone is inhibition of *Plasmodium* and *Pneumocystis* complex III (refs 19, 20, 21). To investigate whether atovaquone also inhibits complex III in human cancer cells, the activity of the ETC complexes I–IV was assessed in FaDu cells permeabilized with digitonin. Digitonin (0.005%) was sufficient to permeabilize the cells, allowing respiration of succinate, a substrate that does not cross intact cell membranes (Supplementary Fig. 2c). Complex I-dependent respiration was significantly inhibited by 30 μM atovaquone, the complex III inhibitor, myxothiazol and the complex I inhibitor, rotenone (Fig. 3a). Complex II-dependent respiration was significantly inhibited by 30 μM atovaquone, myxothiazol and the complex II inhibitor, malonate. Complex III-dependent respiration was significantly inhibited by 30 μM atovaquone, myxothiazol and the complex III inhibitor, antimycin A. Complex IV-dependent respiration was significantly inhibited by the complex IV inhibitor, sodium azide, but not by 30 μM atovaquone, and was less significantly inhibited by myxothiazol. Taken together, these results suggest that the primary mechanism of action of atovaquone in FaDu cells is inhibition of complex III, which then causes a decrease in the activity of the upstream ETC complexes.

To study complex III in isolation from the other ETC complexes, the reduction of excess cytochrome *c* was measured in mitochondria isolated from FaDu cells, a reaction catalysed by complex III. There was a significant decrease in complex II/III activity by the complex II inhibitor, malonate, and by the complex III inhibitors, antimycin, myxothiazol and 30 μM atovaquone (Fig. 3b). Due to the dependence of this assay on complex II activity, a complex II-specific assay was performed in parallel using mitochondria isolated from FaDu cells. There was a significant decrease in complex II activity by the complex II inhibitor, malonate (Fig. 3c). However, there was no inhibition of complex II activity by the complex III inhibitors, antimycin, myxothiazol or 30 μM atovaquone. These findings were also observed in mitochondria isolated from bovine heart (Supplementary Fig. 2d,e). To demonstrate that complex III inhibition is sufficient to reduce the OCR, FaDu cells were incubated with antimycin A or myxothiazol and the OCR was measured for 45 min. A rapid and significant decrease in the OCR was observed in a dose-dependent manner for both inhibitors, with no decrease at 10 nM, but a significant decrease at 1 μM (Fig. 3d,e). Furthermore, incubation of FaDu spheroids for 24 h with 1 μM antimycin or myxothiazol eliminated spheroid hypoxia without affecting spheroid size, whereas no reduction in hypoxia was observed at 10 nM (Fig. 3f,g and Supplementary Fig. 2f). These findings indicate that atovaquone specifically inhibits complex III in FaDu cells, and that complex III inhibition is sufficient to reduce both the OCR and spheroid hypoxia.

### ATO inhibits pyrimidine synthesis

**Figure 4:**
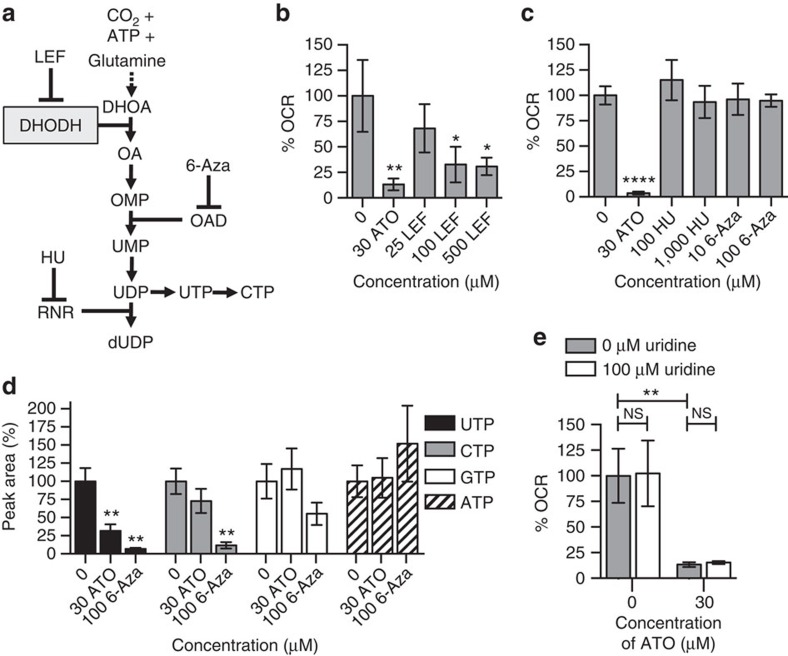
Atovaquone inhibits pyrimidine synthesis. (**a**) The pyrimidine synthesis pathway. 6-Aza, 6-azauridine; DHODH, dihydroorotate dehydrogenase; HU, hydroxyurea; LEF, leflunomide; OAD, orotidylic acid decarboxylase; RNR, ribonucleotide reductase. (**b**) The % OCR following 2 h incubation of FaDu cells with LEF or 30 μM ATO. (**c**) The % OCR following 24 h incubation of FaDu cells with HU, 6-Aza or 30 μM ATO. (**d**) NTP peak area levels relative to the DMSO control and normalized to cell number after 24 h incubation with 30 μM ATO or 100 μM 6-Aza. (**e**) The % OCR following 2 h incubation of FaDu cells with either 100 μM uridine, 30 μM ATO or both 100 μM uridine and 30 μM ATO. % OCR is presented relative to the DMSO control and corrected for cell number (*n*≥3). One-way ANOVA with Bonferroni was performed for all of the experiments apart from **e**, for which an unpaired two-tailed *T*-test was performed (****P<0.0001, ***P<0.001, **P<0.01, *P<0.05). All values are presented as mean±s.d. except for the HPLC data, which are presented as mean±s.e.m. ATO, atovaquone.

Dihydroorotate dehydrogenase (DHODH) is an enzyme of the *de novo* pyrimidine synthesis pathway situated on the inner mitochondrial membrane (Fig. 4a). In biochemical assays, atovaquone inhibits not only complex III, but also DHODH^22^. To investigate whether a known DHODH inhibitor could reduce the OCR, FaDu cells were incubated for 2 h with leflunomide^23^,^24^, which caused a dose-dependent decrease in the OCR (Fig. 4b). However, inhibition of the pyrimidine synthesis pathway downstream of DHODH by the orotidylic acid decarboxylase inhibitor, 6-azauridine (6-Aza), or by the ribonucleotide reductase inhibitor, hydroxyurea^25^,^26^ did not cause a decrease in the OCR of FaDu cells after 24 h incubation (Fig. 4c). The levels of nucleoside triphosphates were measured by high-performance liquid chromatography (HPLC) in FaDu cells treated for 24 h with atovaquone or 6-Aza. In the cells treated with atovaquone, there was a significant decrease in UTP levels, a non-significant decrease in CTP levels, and no significant changes in GTP or ATP levels (Fig. 4d and Supplementary Fig. 2g). Therefore atovaquone appears to specifically affect pyrimidine synthesis. In the cells treated with 6-Aza, there were significant decreases in UTP and CTP levels, a non-significant decrease in GTP levels, and a non-significant increase in ATP levels, indicating that 6-Aza primarily affects pyrimidine synthesis under these conditions. Furthermore, the observed decrease in the OCR induced by incubation with atovaquone for 2 h was not rescued by addition of the pyrimidine precursor, uridine, in FaDu cells (Fig. 4e). These results suggest that inhibition of DHODH by atovaquone causes a decrease in both the OCR and pyrimidine synthesis, but that the decrease in pyrimidine synthesis is unlikely to cause the decrease in the OCR.

### ATO doesn't affect HIF-1α or intrinsic radiosensitivity

HIF-1 is a heterodimeric transcription factor that promotes angiogenesis and increased proliferation^1^. The HIF-1α subunit is rapidly degraded under normoxia, but is stabilized under hypoxia. To assess the effect of atovaquone on HIF-1α expression *in vitro*, FaDu, HCT116 and H1299 cells were incubated with atovaquone for 2 or 24 h in either normoxic (20% O_2_) or hypoxic (0.5% O_2_) conditions. HIF-1α was not stabilized by atovaquone under normoxic conditions and no significant differences in HIF-1α protein levels were observed under hypoxic conditions (Supplementary Fig. 3a,b).

Radiation sensitivity can be affected not only by tumour hypoxia, but also by alteration of the intrinsic radiosensitivity of the cancer cells^1^,^3^,^4^. Therefore colony formation assays were conducted to investigate whether atovaquone alters intrinsic radiation sensitivity in monolayer FaDu, HCT116 or H1299 cells. Assessment of colony formation revealed that atovaquone did not alter radiosensitivity under either normoxia (20% O_2_) or severe hypoxia (<0.1% O_2_) (Supplementary Fig. 3c and Supplementary Table 2). This indicates that, as expected, atovaquone is not an intrinsic radiation sensitizer.

### ATO affects tumour hypoxia and radiation response

**Figure 5:**
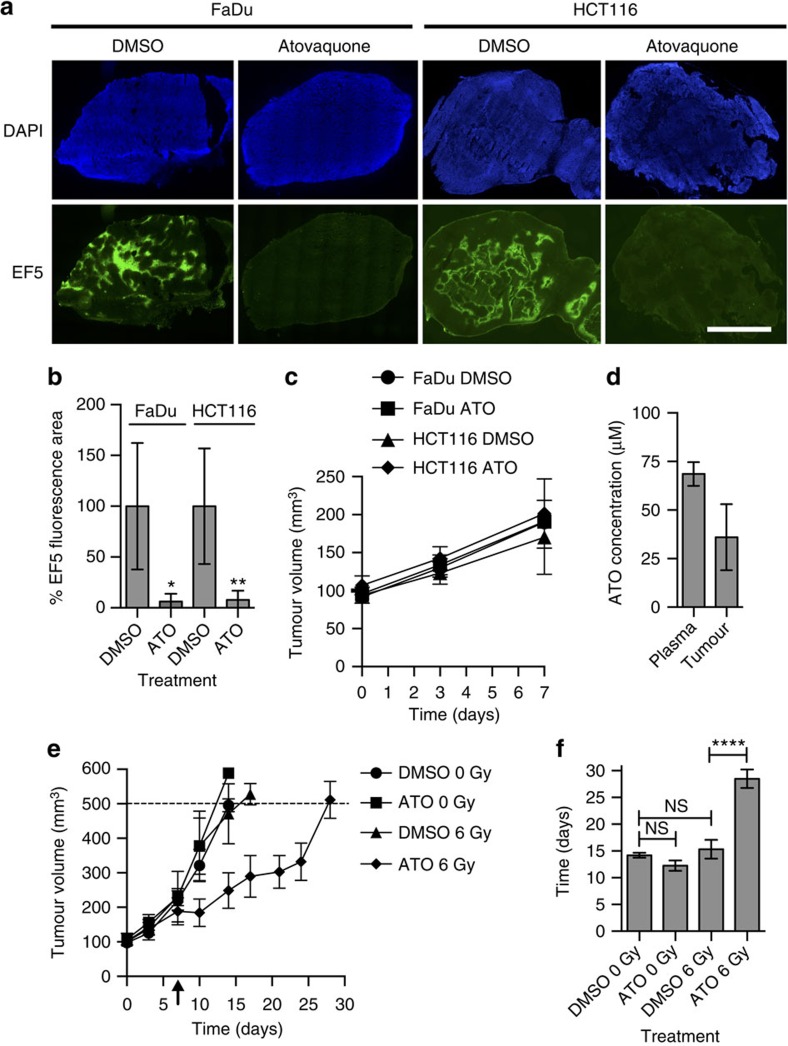
ATO reduces tumour hypoxia and improves tumour radiation response. Mice bearing FaDu and HCT116 xenograft tumours were treated with atovaquone for 7 days, and were injected with EF5 on day 7. (**a**) Immunostaining for EF5 with DAPI as a nuclear counterstain. Scale bar, 2 mm. (**b**) % EF5 fluorescent area relative to the DMSO-treated tumours. (**c**) Average tumour size on days 0, 3 and 7. (**d**) Mean concentration of atovaquone in the blood plasma after 5 days treatment, and mean concentration in the excised HCT116 tumours. (**e**) Mice bearing FaDu xenograft tumours were treated with atovaquone for 10 days, with 6 Gy radiation on day 7 (indicated by arrow). Average tumour volume is shown (mm^3^). One tumour from a mouse treated with atovaquone and radiation took 42 days to reach 500 mm^3^ and was excluded from analysis. (**f**) Average time taken for tumours to reach the maximum volume of 500 mm^3^ (days). At least five mice were used for each treatment group. One-way ANOVA were performed to assess statistical significance with Bonferroni post correction (****P<0.0001, **P<0.01, *P<0.05). All values are presented as mean±s.d. except for the HPLC data, which are presented as mean±s.e.m. ATO, atovaquone.

Given that atovaquone completely alleviated spheroid hypoxia at pharmacologically relevant doses, mice bearing FaDu and HCT116 xenografts were treated with atovaquone for 7 days to investigate the effect on tumour hypoxia. The tumours were harvested on day 7, and EF5 staining confirmed that tumour hypoxia was virtually abolished in both the FaDu and HCT116 xenografts (Fig. 5a,b). As expected, the tumour volume was not significantly altered during the course of the experiment (Fig. 5c). The mean concentration of atovaquone in the blood plasma after 5 days treatment was 68.6 μM, and mean concentration in the HCT116 tumours was 36.0 μM (Fig. 5d).

Given the dramatic decrease in tumour hypoxia observed following atovaquone treatment, radiation response was assessed in mice bearing FaDu xenografts. The mice were treated with atovaquone for 7 days prior to irradiation with a single dose of 6 Gy, and then atovaquone treatment was continued for a further 3 days. In the unirradiated mice, the average time taken for the tumours to reach 500 mm^3^ was 14.2 days for mice treated with DMSO, and 12.3 days for mice treated with atovaquone, a non-significant difference, indicating that atovaquone alone does not alter tumour volume (Fig. 5e,f). In the irradiated mice, the average time taken for the tumours to reach 500 mm^3^ was 15.3 days for mice treated with DMSO, but 28.5 days for mice treated with atovaquone, a significant synergistic growth delay of 13.2 days between the two irradiated groups (*P*<0.0001). One tumour, excluded from analysis, treated with both atovaquone and radiation had an excellent response, reaching 500 mm^3^ 42 days after the initiation of drug treatment. The observed tumour growth delay is likely due to the reduction in tumour hypoxia observed with atovaquone treatment.

### Biguanides affect spheroid hypoxia and radiation response

**Figure 6:**
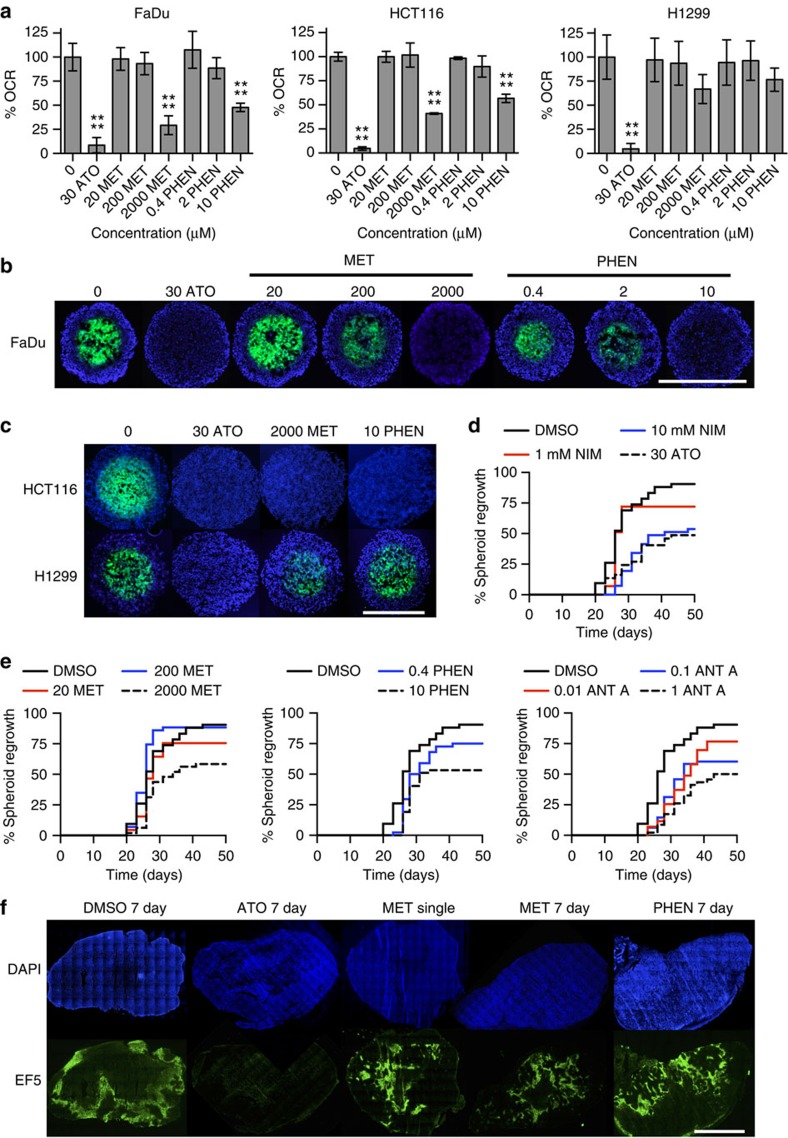
Biguanides alleviate spheroid hypoxia and improve radiation response. (**a**) The OCR of FaDu, HCT116 and H1299 cells were measured after 24 h incubation with atovaquone, metformin or phenformin at the indicated concentrations. The % OCR is shown relative to the DMSO control and normalized to the relative cell number obtained by hoechst staining at the end of the experiment. The data are an average of three independent experiments. One-way ANOVA were performed to assess statistical significance with Bonferroni post correction (mean±s.d., ****P<0.0001). (**b**,**c**) FaDu (**b**), HCT116 and H1299 (**c**) spheroids were treated with DMSO, atovaquone, metformin or phenformin for 24 h at the indicated concentrations (μM). Hypoxia was assessed by staining central spheroid sections for EF5 (green), with DAPI as a nuclear counterstain (blue). Scale bar, 600 μm. Hypoxia was assessed in at least five spheroids per treatment for each experiment (*n*=3), and the data are representative of three independent experiments. (**d**,**e**) FaDu spheroids were treated with DMSO, atovaquone, nimorazole, antimycin A, metformin or phenformin for 24 h at the indicated concentrations (μM). After 24 h the spheroids received 10 Gy, and were then allowed to recover in drug-free medium. Time post treatment is shown (days). One replicate of the experiment was conducted with at least 37 spheroids in each treatment group. (**f**) Mice bearing FaDu xenograft tumours were treated with 50 mg kg^−1^ per day atovaquone for 7 days, 250 mg kg^−1^ metformin single dose, 250 mg kg^−1^ per day metformin for 7 days or 100 mg kg^−1^ per day phenformin for 7 days. EF5 was injected 30 min after the final dose, and then the mice were killed after a further 2 h. Immunostaining for EF5 with DAPI as a nuclear counterstain is shown. Scale bar, 2 mm. Five mice were used for each treatment group. ANT A, antimycin A; ATO, atovaquone; MET, metformin; NIM, nimorazole; PHEN, phenformin.

Previous publications have demonstrated that the anti-diabetic biguanide, metformin, reduces the OCR and tumour hypoxia^11^,^12^. To evaluate the relative efficacy of atovaquone, metformin and its precursor, phenformin, the OCR was measured in FaDu, HCT116 and H1299 cells following 24 h treatment. A significant decrease in the OCR of over 90.0% was observed in all three cells lines treated with 30 μM atovaquone (Fig. 6a). A significant decrease of 70.7% in FaDu cells and 59.0% in HCT116 cells was observed at 2 mM metformin. However, metformin was less potent in H1299 cells, causing a decrease of 33.1% at 2 mM, which was not statistically significant. A significant decrease in the OCR of 52.2% in FaDu cells and 43.3% in HCT116 cells was observed at 10 μM phenformin. Phenformin was also less potent in H1299 cells, causing a non-significant decrease of 23.4%. There were no significant changes in the OCR at 200 or 20 μM metformin, or at 2 or 0.4 μM phenformin in any of the cell lines. Metformin and phenformin did not affect cell survival at any concentration tested (Supplementary Fig. 4a). To investigate the kinetics of this effect in FaDu cells, the OCR was measured for 15 h after injection of atovaquone, metformin or phenformin. The OCR was reduced by 87.3% by atovaquone within 2 h (Supplementary Fig. 4b). However, a slower decrease in the OCR was observed following treatment with 2 mM metformin or 10 μM phenformin, with a reduction of <10% within 2 h and a reduction of 55.9% and 36.0%, respectively, after 10 h.

To assess whether the observed decrease in the OCR translated to a decrease in spheroid hypoxia, FaDu, HCT116 and H1299 spheroids were treated with atovaquone, metformin or phenformin for 24 h, and the hypoxic fraction was evaluated by EF5 staining. There was near-complete loss of hypoxia in FaDu and HCT116 spheroids at 30 μM atovaquone, 2 mM metformin and 10 μM phenformin (Fig. 6b,c, Supplementary Fig. 4c). In contrast, there were non-significant decreases of 41.8% and 9.8% in H1299 spheroids following treatment with 2 mM metformin and 10 μM phenformin, respectively. No significant decreases in hypoxia were observed at 20 μM or 200 μM metformin, or at 0.4 μM or 2 μM phenformin in FaDu spheroids. No treatments caused a significant change in spheroid diameter (Supplementary Fig. 4d). These results are in accordance with the OCR results shown in Fig. 6a, suggesting that a significant reduction in the OCR by metformin and phenformin is required to reduce spheroid hypoxia.

Nimorazole is a 2-nitroimidazole that acts as an oxygen mimetic, which has previously been shown to improve loco-regional control when combined with radiotherapy in head and neck cancer^27^,^28^. To compare the ability of nimorazole, atovaquone, metformin, phenformin and antimycin A to improve radiation response, FaDu spheroids were treated for 24 h with these drugs, irradiated at 10 Gy, and then allowed to recover in fresh medium. Fifty days after irradiation, 90.5% of the DMSO-treated spheroids were reformed (Fig. 6d). In comparison, the percentage reformation of spheroids for the highest concentration of each treatment was 53.7% for 10 mM nimorazole, 48.6% for atovaquone, 58.3% for 2 mM metformin, 53.2% for 10 μM phenformin, and 50.5% for 1 μM antimycin A (Fig. 6d,e). A less-dramatic decrease in spheroid regrowth was observed at the lower concentrations of the drugs, with a percentage spheroid reformation of 72.1% for 1 mM nimorazole, 75.6% for 20 μM metformin, 75.0% for 0.4 μM phenformin and 60.4% for 0.1 μM antimycin A. For unirradiated spheroids, no drug treatment caused a significant decrease in spheroid growth compared with the DMSO control after 35 days (Supplementary Fig. 5a,b). These results demonstrate that the greatest improvement in radiation response is observed at concentrations of the drugs that eliminate spheroid hypoxia, and that this is sufficient to improve radiation response in a manner comparable to 10 mM nimorazole.

Having demonstrated that metformin and phenformin reduce hypoxia in spheroids, their effect on hypoxia was next examined *in vivo*. Mice bearing FaDu xenografts were treated with DMSO for 7 days, 50 mg kg^−1^ atovaquone for 7 days, a single dose of 250 mg kg^−1^ metformin, 250 mg kg^−1^ per day metformin for 7 days or 100 mg kg^−1^ per day phenformin for 7 days. The tumours were harvested 2.5 h after the final dose of drug, and EF5 staining was performed. Atovaquone caused a significant 73.7% reduction in hypoxia, and 7 days metformin treatment caused a 43.1% decrease in hypoxia, although this was not statistically significant. There were no significant changes in hypoxia following a single dose of metformin or 7 days phenformin (Fig. 6f and Supplementary Fig. 5c). After a single dose of metformin the mean plasma concentration was 109 μM, and mean tumour concentration was 151 μM (Supplementary Fig. 5d).

## Discussion

Our screen, designed to find compounds that decrease tumour hypoxia by inhibiting oxygen consumption, identified atovaquone. Atovaquone caused a rapid decrease in oxygen consumption in a wide range of cancer cell lines. It virtually abolished both spheroid hypoxia and tumour hypoxia, and augmented the effects of radiotherapy. Its mechanism of action is likely the inhibition of the mitochondrial cytochrome *bc*_1_ complex (complex III).

The efficacy of radiotherapy can be improved by the reoxygenation of radioresistant hypoxic tumour cells^1^,^3^,^4^. This study reveals that atovaquone inhibits oxygen consumption, spheroid hypoxia and tumour hypoxia, but has no effect on intrinsic radiosensitivity *in vitro* under normoxic or hypoxic conditions. However, atovaquone improved radiation sensitivity in both spheroids and xenograft tumours. Therefore we propose that the radiation sensitization following atovaquone treatment could be due to a decrease in hypoxia rather than an increase in intrinsic radiosensitivity. It is not expected that atovaquone would exacerbate radiation-induced side effects in a clinical setting because irradiated normal tissues are not generally hypoxic. The improvement in radiation response in spheroids was comparable to 10 mM nimorazole, although the percentage reformation of spheroids was 72.1% for 1 mM nimorazole, compared with 48.6% for atovaquone, and the concentration of nimorazole in the blood plasma of patients is in the sub-millimolar range^29^,^30^,^31^. This indicates that the improvement of radiation response in spheroids is superior for atovaquone compared with nimorazole at pharmacological concentrations.

Atovaquone is FDA approved as a single agent to treat pneumocystis pneumonia, which is caused by *Pneumocystis jirovecii*^18^. It is also used, primarily in combination with proguanil, as chemoprophylaxis against malaria, or to treat malaria, which is caused by *Plasmodium falciparum*. Atovaquone has a good safety profile with only mild side effects, is well absorbed, can accumulate in tissues, is eliminated by the liver after 50–84 h, and is not metabolized *in vivo* to a significant extent^18^,^32^,^33^,^34^. It is an analogue of the electron carrier, ubiquinone, which is reduced to ubiquinol by DHODH, complex I and complex II, and is then oxidized back to ubiquinone by complex III (ref. 18). In parasites, the primary mechanism of action of atovaquone is inhibition of complex III (refs 20, 21). In biochemical assays, atovaquone inhibits *P. falciparum* complex III with an IC_50_ of 3 nM, but is far less potent against human complex III, which it inhibits with an IC_50_ of 70–460 nM (refs 20, 21). It was previously assumed that this comparably low efficacy against purified human complex III would not translate to the inhibition of this enzyme *in vitro*. However, several experiments conducted in this study indicate that atovaquone inhibits complex III in cancer cells. First, the OCR is inhibited by atovaquone under conditions promoting complex III-dependent respiration in digitonin-permeabilized cells. Second, the reduction of cytochrome *c* is inhibited by atovaquone under conditions promoting complex III-dependent respiration in isolated mitochondria, but the reduction of dichlorophenolindophenol by complex II is not inhibited by atovaquone. Furthermore, complex III inhibition by atovaquone, antimycin A or myxothiazol is sufficient to markedly reduce the OCR and consequently to eradicate spheroid hypoxia and improve radiation response.

Complex III and DHODH, an enzyme of the *de novo* pyrimidine synthesis pathway, are situated in the inner mitochondrial membrane in higher eukaryotes, and their activities are closely linked. Cellular DHODH activity is inhibited by ETC agonists such as antimycin A and nitric oxide, and by DHODH agonists such as leflunomide and brequinar, but only DHODH agonists inhibit the activity of purified DHODH^24^,^35^,^36^. Atovaquone is a much more potent inhibitor of purified human complex III (IC_50_=70–460 nM) than human DHODH (IC_50_=14.5 μM), suggesting that it primarily acts as a complex III inhibitor in cancer cells^20^,^21^,^22^. For these reasons, it is our conjecture that atovaquone primarily inhibits complex III, which causes depletion of the DHODH cofactor, ubiquinone, and subsequent inhibition of DHODH activity and downstream pyrimidine synthesis.

The anti-diabetic drug metformin, reduces the OCR by inhibition of complex I, and decreases tumour hypoxia^11^,^12^. This study demonstrated that metformin and phenformin reduce the OCR in FaDu and HCT116 cells, eliminate hypoxia in FaDu and HCT116 spheroids, and elicit an improved radiation response. In contrast, metformin and phenformin were ineffective in reducing the OCR in H1299 cells, or in alleviating H1299 spheroid hypoxia. In comparison, the efficacy of atovaquone is very similar in a wide range of tumour cell lines, and it eliminates hypoxia in FaDu, HCT116 and H1299 spheroids.

In accordance with a previous publication^12^, this study demonstrated a decrease in tumour hypoxia following 7 days metformin treatment, albeit non-significant. The reason that metformin eliminated spheroid hypoxia but caused a less significant reduction in tumour hypoxia could be due to a discrepancy between the dose required to elicit this effect in spheroids and the concentration achieved in tumours. There is an ongoing debate concerning the doses of metformin used in murine cancer models, and whether they are clinically relevant^37^,^38^. Diabetics treated with the standard clinical doses of 1,500–2,500 mg per day metformin have maximum plasma levels in the range of 10–25 μM, and non-diabetic cancer patients receiving 1,500–2,000 mg per day have maximum plasma levels of 2.8–7 μM (refs 38, 39). In comparison, the maximum plasma levels achieved in murine models following oral administration of 125–350 mg kg^−1^ per day metformin for 2 weeks are 5–47 μM, with a level of metformin in the tumours of 32–200 μM (refs 37, 38). At first glance, this may make the observed effects of metformin on the OCR and spheroid hypoxia at 2 mM appear irrelevant both *in vivo* and in the clinic. However, several explanations have been proposed to explain this discrepancy^12^,^37^,^38^. These include the predicted accumulation of metformin in the mitochondria over time to achieve doses required for complex I inhibition, the effects of the tumour microenvironment, and a lower concentration of glucose, serine and glutamine in the tumour, metabolites that have been shown to cause resistance to metformin *in vitro*. It is possible that the dose of metformin could be raised to achieve higher plasma concentrations in cancer patients, but this may increase potentially serious adverse effects such as lactic acidosis. Indeed, phenformin was withdrawn from clinical use in the 1970s due to a high risk of lactic acidosis. This may preclude its use as an anti-cancer therapeutic, although it is possible that a good therapeutic ratio exists for phenformin at lower doses.

In contrast to metformin, the concentration of atovaquone required to reduce the OCR *in vitro* and to alleviate tumour hypoxia *in vivo* is achievable in patients. It reaches a steady-state concentration in the blood plasma of 65 μM or more after 1–2 weeks administration in pneumocystis pneumonia patients at doses that are used routinely in the clinic, but reduces the OCR by up to 90% within 2 h (refs 32, 33). The mean concentration of atovaquone in the mouse blood plasma after 5 days treatment was 68.6 μM, suggesting that doses required to reduce tumour hypoxia could be achieved without toxicity in patients.

Although atovaquone did not have a significant effect on the cell number after 4.5 h, there was a significant decrease in the cell number after 24 h in monolayer cells. We do not believe that this finding effects the interpretation of any of our data or the potential clinical applicability of atovaquone because it reduces the OCR within 2 h without a significant effect on cell number, it does not effect the growth of spheroids or xenograft tumours in the absence of radiation, and it has an excellent clinical safety profile.

Some studies have described cycling hypoxia in tumours, characterized by periods of hypoxia followed by periods of reoxygenation, which can lead to excessive ROS production and may cause increased resistance to therapy^40^,^41^. This phenomenon is thought to occur as a result of changes in vessel perfusion and fluctuations in the flux of red blood cells within perfused vessels^40^. However, the contribution of tumour cells that undergo cycling hypoxia to poor patient outcome is currently unclear. Reoxygenation has also been shown to increase the potential risk of metastasis^42^,^43^. These studies highlight potential issues with reoxygenating tumours. However, the literature overwhelmingly suggests that the benefits of eradicating hypoxia outweigh any theoretical risks, with a recent meta-analysis supporting hypoxia modification as a strategy to improve response to therapy in head and neck squamous cell carcinoma^1^,^3^,^4^.

This study suggests that attenuation of tumour hypoxia by atovaquone may be a highly appealing clinical strategy to increase the sensitivity of tumour cells to radiation in multiple different tumour types. Accordingly, we are now initiating a proof of principle, ‘window of opportunity' clinical trial to investigate whether atovaquone reduces tumour hypoxia in lung cancer patients prior to surgical resection, on the basis of imaging, serological markers and immunohistochemical markers.

## Methods

### Cell lines and compounds

All cell lines were purchased from the American Type Culture Collection (ATCC). STR profiling (DNA Diagnostics Centre, UK) and mycoplasma testing (Lonza) are conducted routinely for these cell lines. Cells were cultured at 37 °C with 5% CO_2_ in Dulbecco's modified Eagle's medium (DMEM, Sigma) supplemented with 10% fetal bovine serum (FBS, Lonza). All compounds were obtained from Sigma except duroquinol (Tokyo Chemical Industry), and EF5, a gift from Cameron Koch's Laboratory, University of Pennsylvania). All compounds were prepared from powder. For the experiments under hypoxic conditions, cells were grown at 0.5% O_2_ in an *in vivo* 2400 Ruskinn chamber (Biotrace Fred Baker), and at <0.1% O_2_ in a Bactron II chamber (Shell Labs).

### OCR primary screen

FaDu cells were incubated with either the Pharmakon 1,600 (Microsource) or the 97-compound TDI Oncology Drug Set (Target Discovery Institute, Oxford University) compound libraries at 2 and 10 μM for 24 h in DMEM containing 5 mM glucose and 4 mM L-glutamine. The mitochondrial-specific OCR was determined by taking a basal OCR measurement in XF assay medium (Seahorse Biosciences) containing 5 mM galactose, 5 mM sodium pyruvate and 4 mM L-glutamine using an XF96 Analyzer (Seahorse Biosciences) and subtracting the OCR measured following injection of 2 μM antimycin A. The cells were then fixed in ice-cold methanol for 5 min, and incubated in 1 × PBS, 4 μg ml^−1^ Hoechst 33258 (Sigma) for 30 min before measuring fluorescence using a POLARstar Omega plate reader (BMG Labtech) to obtain the relative cell number per well. The mitochondrial-specific OCR was corrected using the relative cell numbers, and compounds that caused a reduction in cell number of more than 66% compared with the DMSO control wells for each plate were excluded. Two repetitions of the screen were conducted. The *Z* scores were generated and used to rank the compounds for each repeat, and the rank product was used to sort the compounds. The compounds with the top 130 rank products were chosen for the secondary screen.

### OCR secondary screen

The secondary screen was conducted using the same protocol as the primary screen, except FaDu cells were incubated with 80 nM, 400 nM, 2 μM and 10 μM of the compounds, the assay medium contained 5 mM glucose, 5 mM sodium pyruvate and 4 mM L-glutamine, and three replicates were conducted. The analysis was conducted as described for the primary screen, using the OCR score to rank the compounds for each repeat. The OCR score is defined as the ‘OCR normalized to cell number'−‘Average of OCR normalized to cell number for the DMSO wells for each plate'/‘s.d. of OCR normalized to cell number for the DMSO wells for each plate'. The list was filtered to include only compounds approved for systemic use in humans, excluding those approved for topical or veterinary use. The rank product was used to sort the compounds. Compounds were excluded based on prospectively defined criteria: OCR, cell viability, pharmacokinetics, FDA-approved use and known hypoxia modification.

### OCR

The cells were grown for 24 h in DMEM containing 5 mM glucose and 4 mM L-glutamine, and then measurements of the OCR were taken in XF assay medium (Seahorse Biosciences) containing 5 mM glucose, 5 mM sodium pyruvate and 4 mM L-glutamine using an XF96 Analyzer (Seahorse Biosciences). For the kinetics experiments, measurements were collected every 15 min following injection of atovaquone. For the experiments with uridine and pyrimidine synthesis inhibitors, the cells were incubated with the compounds for 2 or 24 h as indicated, the medium was replaced with the assay medium also containing the compounds, and then measurements were made. The OCR measurements were corrected for cell number using Hoechst staining, as described above. For the experiments using permeabilized FaDu cells, 0.005% digitonin was the permeabilization agent, and the % OCR was measured within 15 min of its addition. Permeabilization of the plasma membrane allows the substrates respired by the mitochondria to be dictated by the added medium, and allows non-membrane permeable substrates to access the mitochondria^44^. The activities of complexes I and II were assayed using substrates specific to each complex in conjunction with an inhibitor of the other complex: 5 mM pyruvate with 1 mM malic acid to promote complex I-dependent respiration, and 10 mM succinate with 1 μM rotenone to promote complex II-dependent respiration. Complex III-dependent respiration was assayed using 500 μM duroquinol as a substrate. Complex IV-dependent respiration was assayed using 500 μM *N*,*N*,*N*′,*N*′-tetramethyl-*p*-phenylenediamine (TMPD) as a substrate, in conjunction with 2 mM ascorbate to maintain TMPD in reduced form. The inhibitors used for the assay were 2 μM rotenone for complex I, 10 mM malonate or 1 mM malic acid for complex II, 2 μM myxothiazol or 2 μM antimycin A for complex III, and 20 mM sodium azide for complex IV.

### Spheroids

FaDu spheroids were grown using the liquid overlay technique, and HCT116 and H1299 spheroids were grown in lipidure-coated U-bottom plates (UMS Bio). Half of the medium was replaced twice per week. Spheroid hypoxia was quantified by EF5 staining, using an anti-EF5 antibody (both reagents obtained from University of Pennsylvania)^7^. Two hundred micromolar EF5 was added to the spheroids for 6 h before fixation in 4% paraformaldehyde for 24 h, incubation in 30% sucrose for 2.5 h, and then addition of OCT (VWR, TissueTek) for cryosectioning. Spheroid sections were incubated for 40 min in TNB blocking reagent (Perkin Elmer), washed for 5 min in 1 × PBS, 0.3% Tween 20, and then incubated overnight with 70 μl of undiluted 75 μg ml^−1^ anti-EF5 antibody. Three washes of 45 min were then performed using 1 × PBS, 0.3% Tween 20 before addition of DAPI vectorshield mounting medium (Vector Laboratories) and fluorescence microscopy (Nikon 90i, Nikon). Spheroid diameter was derived from the spheroid cross-sectional area measured by the Gelcount colony counter (Oxford Optronix) or Axiovert200M (Carl Zeiss MicroImaging GmbH).

### Complex II/III and complex II assays

The complex II/III and complex II assays were performed according to the manufacturer's instructions (Cayman Chemicals), using either mitochondria isolated from FaDu cells or bovine heart mitochondria (Cayman Chemicals). The complex III assay uses the complex II substrate, succinate, to allow generation of the complex III substrate, ubiquinol, by complex II. One micromolar rotenone and 1 mM cyanide were added to inhibit the activities of complexes I and IV, respectively, so the activities of complexes II and III were assayed. The complex II assay uses succinate as a substrate for complex II, and measures reduction of the redox dye, dichlorophenolindophenol, by the ubiquinol generated by complex II. One micromolar rotenone, 10 μM antimycin A and 1 mM cyanide were added to inhibit the activities of complexes I, III and IV, respectively, so the activity of complex II was assayed in isolation. For isolation of mitochondria, FaDu cells were resuspended in 1 ml homogenization buffer at 4 °C: 10 mM Tris, 0.1 mM EDTA, 11.5% sucrose, 1% complete mini protease inhibitor cocktail (Roche), pH 7.4 (ref. 47). The cells were snap frozen on dry ice and then thawed at 37 °C ten times, centrifuged at 750*g*, resuspended in 500 μl homogenization buffer, and centrifuged again, collecting the supernatants at each stage. Finally the pellet was resuspended in 100 μl homogenization buffer omitting EDTA, and stored at −80 °C.

### High-performance liquid chromatography

To quantify NTPs, the cells were lifted and lysed in 100 μl of 6% TCA (ref. 48). Nucleotides were neutralized by extracting the samples with 100 μl of freon (1,1,2 trichlorotrifluorethane): trioctylamine (4:1). The aqueous upper layer was removed and then 40 μl was injected. Chromatography was performed on a Waters 2695 system with diode array detection (Waters 2996), monitoring at 254 nm, with an Ace column (3 μm, 3 × 125 mm) maintained at 30 °C. The samples were maintained at 10 °C. Separation was attained with eluent A (10 mM potassium dihydrogen phosphate, 10 mM tetrabutylammonium hydroxide, 10% methanol, pH 6.9) and eluent B (50 mM potassium dihydrogen phosphate, 6 mM tetrabutylammonium hydroxide, 30% methanol, pH 7), using a flow rate of 0.6 ml min^−1^ and a gradient of 25–80% B over 20 min, and a run time of 25 min. Nucleotides were quantitated against commercially available dNTPs. Peak areas are presented relative to the DMSO control and normalized to the cell number obtained by a Sceptre cell counter (Millipore). Plasma and tumour atovaquone concentrations were determined using lapachol as an internal standard^45^ by negative electrospray ionization on a Waters EMD1000. HPLC was performed on a Cortecs C18 column, 100 × 3 mm (Waters), with eluents of 5 mm ammonium formate in 50% acetonitrile (A) and acetonitrile (B) using a gradient of 10–100% B in 5 min. The flow rate was 0.5 ml min^−1^. Plasma and tumour metformin concentrations were determined using HPLC with absorbance detection at 330 nm using phenformin as an internal standard^46^. Tumours were weighed and homogenized in four volumes of water. To 20 μl plasma or tumour homogenate was added 20 μl phenformin (25 μM), 10 μl 50 mM HCl and 0.5 ml acetonitrile. Samples were centrifuged and the supernatant dried down in a heated centrifugal evaporator. Samples were reconstituted in 100 μl Synergy water. HPLC was performed on a Gemini C18 column, 150 × 3 mm, 3 μm (Phenomenex) at 35 °C, with eluents of 10 mM ammonium bicarbonate pH 10.4 (A) and acetonitrile (B) using isocratic elution of 60% B and a flow rate of 0.4 ml min^−1^.

### Animal models

The project licence covering the animal work (PPL30/2922) was approved by the Oxford University Animal Welfare and Ethical Review Body (AWERB) and granted by the UK Home Office Animals in Science Regulation Unit (ASRU) under the Animals (Scientific Procedures) Act 1986 (ASPA)). FaDu (1 × 10^6^) or HCT116 (5 × 10^6^) cells were inoculated subcutaneously with matrigel (BD Biosciences) in athymic BALB/c nude female mice at age 55–70 days. Once the tumours had reached 100 mm^3^, atovaquone was administered in the drinking water at 50 mg kg^−1^ per day with 2% DMSO and 0.1% carboxymethylcellulose to reflect the clinical administration of atovaquone as an oral suspension^18^,^32^,^33^, assuming that a 20 g mouse would consume 5 ml water per day. Mice were given this dose for 7 days because atovaquone takes 1–2 weeks to reach a steady-state plasma concentration in patients^32^,^33^. Tumours were harvested after i.p. administration of 0.01 ml g^−1^ body weight of 10 mM EF5 after 7 days treatment. Tumours were measured with calipers. For the radiation treatments, a single dose of 6 Gy was given on day 7 with a 250-kV orthovoltage irradiator (Philips RT 250) at a dose rate of 2.63 Gy min^−1^, using copper shielding. Necrosis was quantified by inspection of Hematoxylin and Eosin (H&E) stained sections. Animals were randomly assigned to each treatment group on the day on which their tumours reached 100 mm^3^ using a random number generator (Excel), but the experiment was not blinded. A sample size of five mice per group was used and has 90% power (s.d.=30%, alpha=0.05) to detect a 70% decrease, based on our previous experience and published data^5^,^7^. A one-tailed test was performed for this determination. Use of five animals per treatment group allowed us to overcome the problem of differences in variance between groups being statistically compared. One tumour treated with both atovaquone and radiation had an excellent response, reaching 500 mm^3^ 42 days after the initiation of drug treatment, and was excluded from our analyses.

### Immunoblotting

Protein lysates were made using RIPA lysis buffer (Thermo Scientific). SDS–PAGE electrophoresis was conducted followed by immunoblotting. The primary antibodies used were anti-HIF-1α (1:500, clone 54, BD Biosciences) and anti-GAPDH (1:1,000 6C5, Novus Biologicals). The Alexa Fluor 680 secondary antibody (1:10,000, LICOR) was detected using the Odyssey imaging system (LICOR).

### Colony formation assay

Cells were plated, incubated at 20% O_2_ for 4 h, and then incubated at either 20% O_2_ or <0.1% O_2_ for 6 h with atovaquone prior to irradiation by a ceasium-137 irradiator (Gamma Service: GSR D1; dose rate 1.94 Gy min^−1^). Half an hour after irradiation, all cells were placed at 20% O_2_ for a further 18 h before medium replacement. Colonies were stained with crystal violet and counted using the Gelcount colony counter (Oxford Optronix).

### Statistics

Statistical analysis was performed with Prism 5 software (GraphPad Software Inc.). All values are presented as mean±s.d. except for the colony formation assays and HPLC data, which are presented as mean±s.e.m. Unpaired two-tailed *T*-tests were performed for the uridine and colony formation assays, and for the cell survival assays in Supplementary Fig. 1c. For all other experiments, one-way ANOVAs were performed to assess statistical significance with Bonferroni post correction. *P* values <0.05 were considered significant. All *in vitro* experiments were repeated at least three times.

### Data availability

The data supporting the findings of this study are contained within the Article and Supplementary Information files or available from the corresponding authors upon request.

## Additional information

**How to cite this article:** Ashton, T. M. *et al.* The anti-malarial atovaquone increases radiosensitivity by alleviating tumour hypoxia. *Nat. Commun.* 7:12308 doi: 10.1038/ncomms12308 (2016).

## Supplementary Material

Supplementary Information Supplementary Figure 1-5 and Supplementary Table 1-2 and Supplementary References.

Supplementary Data 1 The OCR screen results.

## Figures and Tables

**Table 1:** The highest ranked compounds that reduce the OCR of FaDu cells at 10 **μ**M.

**Rank**	**Compound**
1	Pyrvinium
2	Berberine
3	Niclosamide
4	Acriflavinium
5	Sorafenib
6	Emetine
7	Plicamycin
8	Suloctidil
9	Pentamidine
10	Amsacrine
11	Phenformin
12	Irinotecan
13	Itraconazole
14	Mitomycin
15	Atovaquone
16	Hydroxyprogesterone
17	Cyclosporine
18	Fenofibrate
