# Supplementary material for: The anti-malarial atovaquone increases radiosensitivity by alleviating tumour hypoxia
Source: Nat Commun. 2016 Jul 25;7:12308. doi: 10.1038/ncomms12308 (PMC4962491; doi:10.1038/ncomms12308)
Supplement: Supplementary Information — Supplementary Figure 1-5 and Supplementary Table 1-2 and Supplementary References. [file ncomms12308-s1.pdf]

Supplementary Information

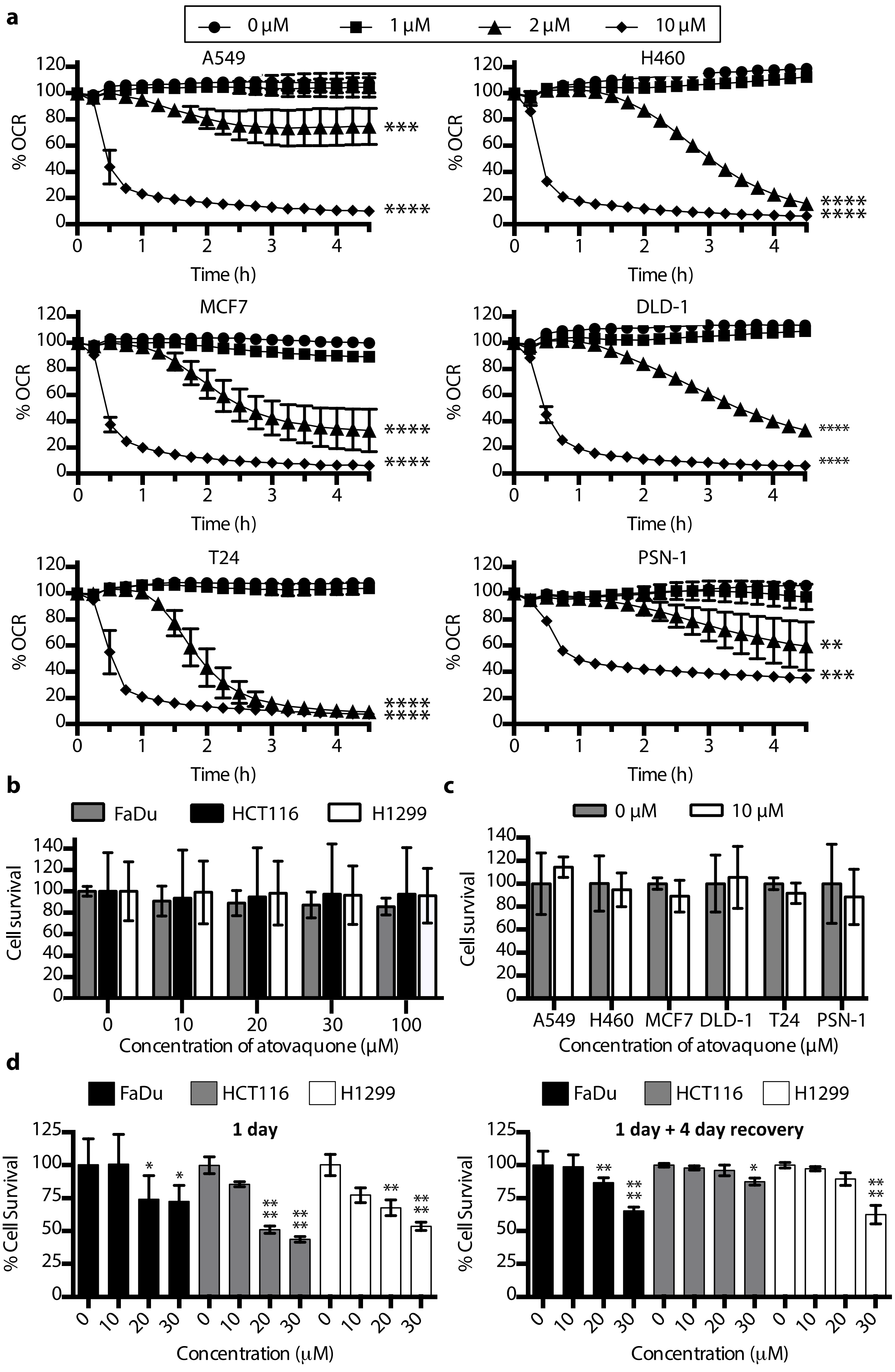
a
0 μM
1 μM
2 μM
10 μM
A549
H460
MCF7
DLD-1
T24
PSN-1
% OCR
Time (h)
***
****
**
b
FaDu
HCT116
H1299
Cell survival
Concentration of atovaquone (μM)
c
0 μM
10 μM
A549 H460 MCF7 DLD-1 T24 PSN-1
d
1 day
1 day + 4 day recovery
% Cell Survival
Concentration (μM)

Supplementary Figure 1 │ Atovaquone decreases the OCR in a range of cancer cells. (**a**) Experiments as described in Fig. 1c, but in A549, H460, MCF7, DLD-1, T24 and PSN-1 cells. % OCR is presented relative to the DMSO control and corrected for cell number. (**b** and **c**) The cells were incubated with atovaquone for 4.5 hours, and then the relative cell number was determined by hoechst staining. Data is presented relative to the DMSO control (n=3). (**d**) The cells were either incubated with atovaquone for 24 hours (left panel) or were incubated with atovaquone for 24 hours, washed, and then allowed to recover in drug-free medium for 4 days (right panel). The relative cell number was determined by hoechst staining. Data is presented relative to the DMSO control (n=3). One-way ANOVA were performed to assess statistical signficance with Bonferroni post correction (Mean±SD, **** $P<0.0001$, *** $P<0.001$, ** $P<0.01$, * $P<0.05$).

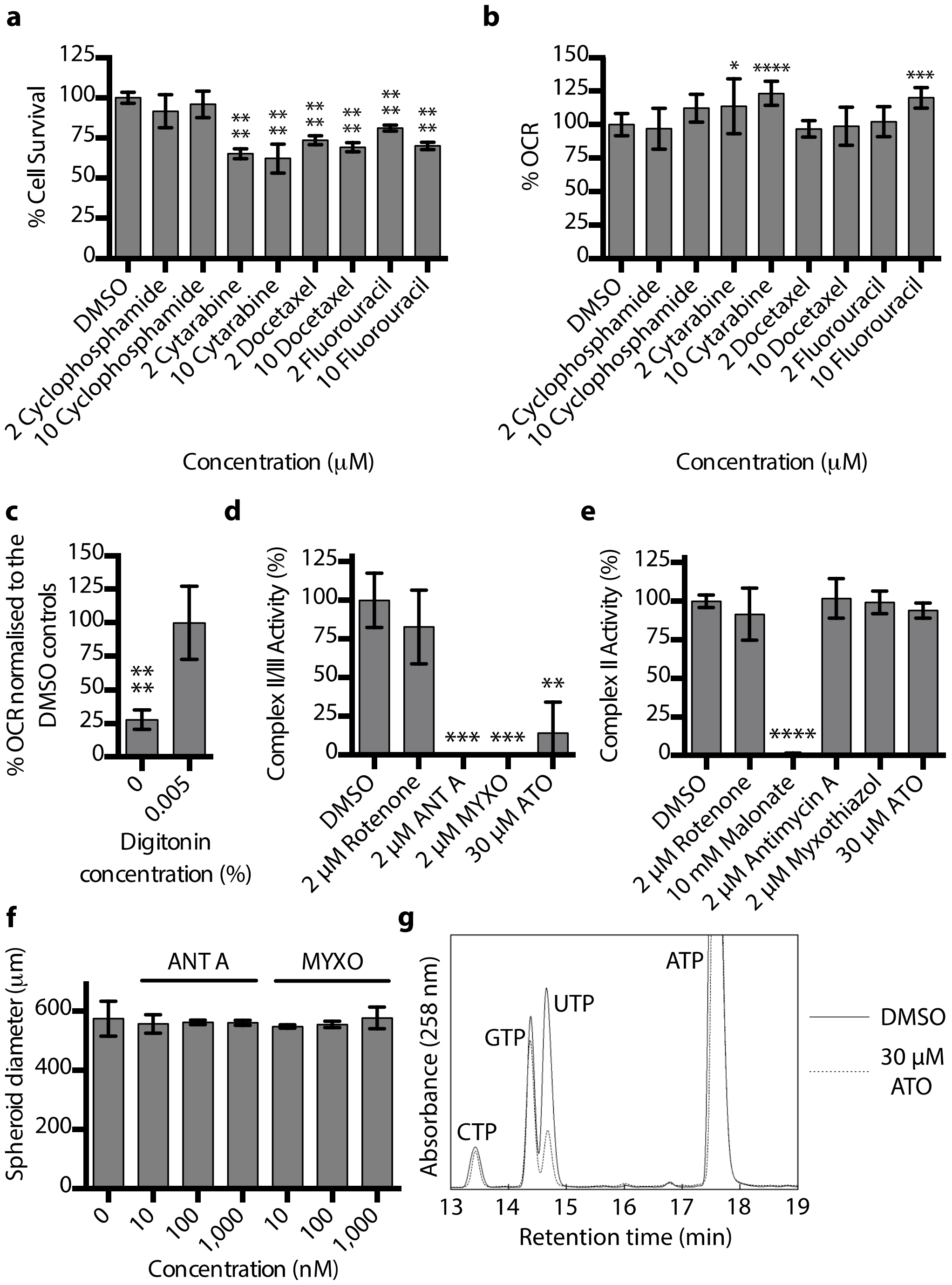

Supplementary Figure 2 | Atovaquone inhibits complex III and pyrimidine synthesis. (**a**) Selected data from the primary screen described in Fig. 1a. The cells were incubated with cyclophosphamide, cytarabine, docetaxel or 5-fluorouracil for 24 hours at 2 μM or 10 μM, as indicated. The relative cell number was determined by hoechst staining. Data is presented relative to the DMSO control (n=4). (**b**) Experiment as described in Supplementary Fig. 2a. % OCR is presented relative to the DMSO control and corrected for cell number. (**c**) 0.005%

digitonin permeabilises FaDu cells, allowing succinate-dependent respiration. The % OCR was measured immediately after permeabilisation. (**d**) Complex II/III activity was measured in bovine heart mitochondria 15 minutes after compound addition. (**e**) Complex II activity was measured in bovine heart mitochondria 15 minutes after compound addition. (**f**) Average spheroid diameter for the experiment described in Fig. 3, f and g. (**g**) A sample chromatogram from the experiment described in Fig. 4d. One-way ANOVA with Bonferroni was performed for all of the experiments (Mean±SD, **** P<0.0001, *** P<0.001, ** P<0.01, * P<0.1).

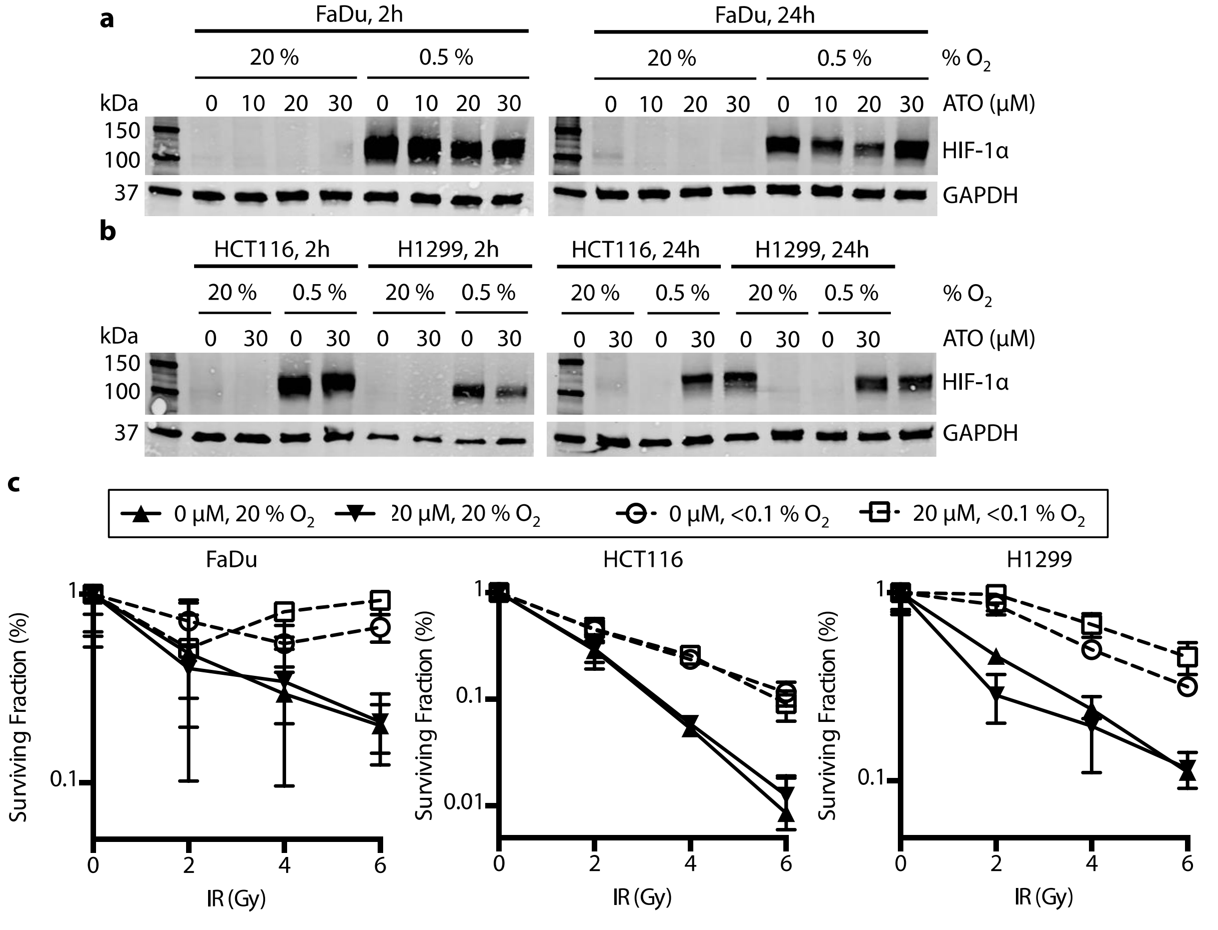

Supplementary Figure 3 | ATO does not affect HIF-1α or intrinsic radiosensitivity. (**a** and **b**) FaDu, HCT116 or H1299 cells were incubated with 0 μM, 10 μM, 20 μM or 30 μM atovaquone (ATO) for 2 hours or 24 hours at either 0.5 % or 20 % oxygen, as indicated. HIF-1α expression was evaluated by western blotting. (**c**) Colony formation assays using

FaDu, HCT116 and H1299 cells. The cells were irradiated after 6 hours incubation at 20 % or <0.1 % O_2 in 0 μM or 20 μM atovaquone, incubated at 20 % O_2 for a further 18 hours, and then washed to remove the atovaquone. All data is representative of 3 independent experiments. No significant sensitisation was observed at any radiation dose (Mean±SEM, Two-tailed unpaired T Test with Welch's correction).

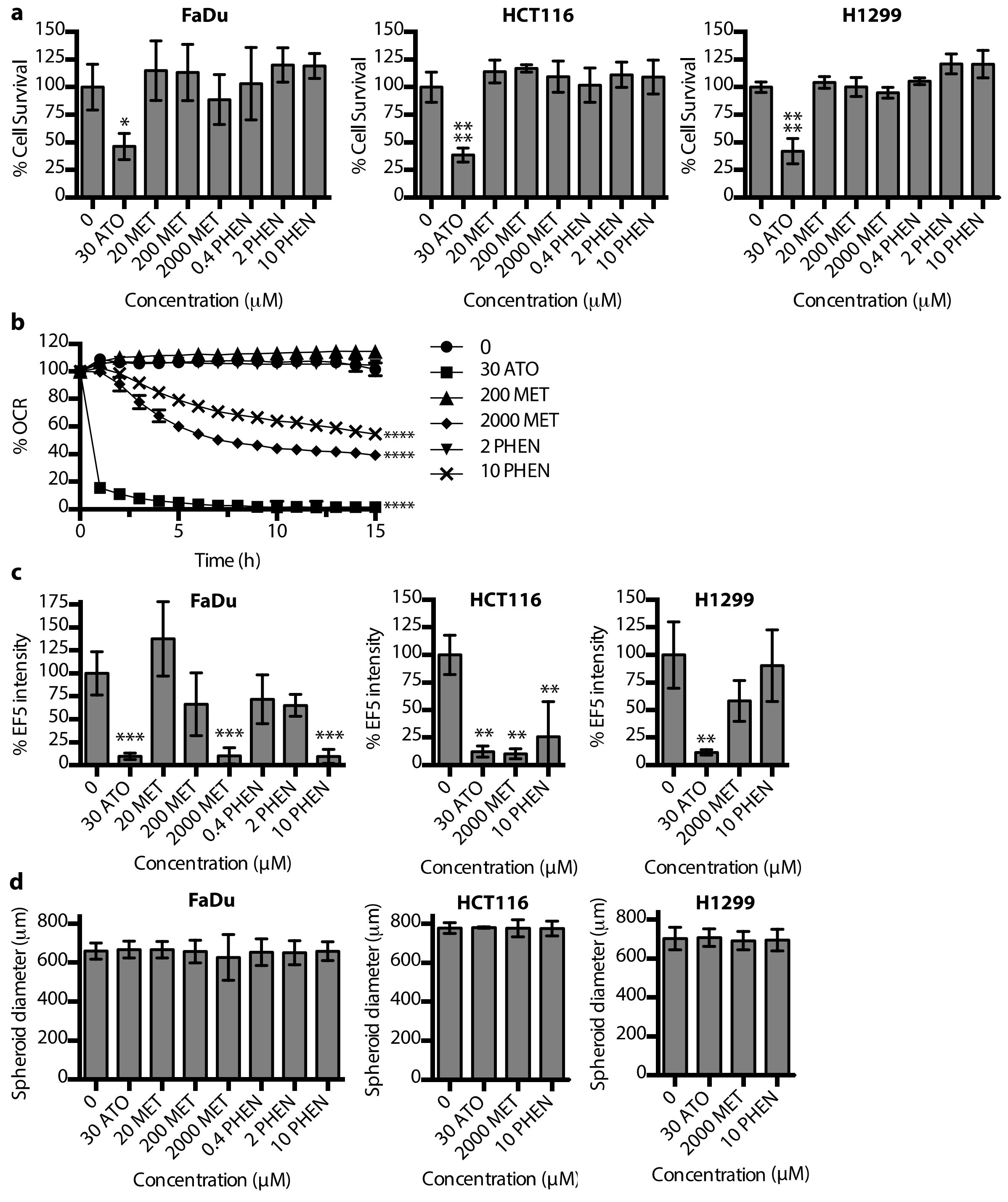

Supplementary Figure 4 | Biguanides reduce the OCR and alleviate spheroid hypoxia.

(**a**) Experiment as described in 6a. The cells were incubated with atovaquone, metformin or phenformin for 24 hours, and then the relative cell number was determined by hoechst staining. Data is presented relative to the DMSO control (n=3). (**b**) The OCR of FaDu cells was measured for 15 hours after injection of atovaquone, metformin or phenformin. The %

OCR post injection is shown relative to the DMSO control and normalised to the relative cell number obtained by hoechst staining at the end of the experiment. (**c**) Experiment as described in 6b and c. % Mean EF5 fluorescence intensity is presented relative to the DMSO controls. (**d**) Experiment as described in 6b and c. Average spheroid diameter (μm). All data is an average of 3 independent experiments apart from (**b**), which is representative of 3 independent experiments. One-way ANOVA were performed to assess statistical with Bonferroni post correction (Mean±SD, **** P<0.0001, *** P<0.001, ** P<0.01, * P<0.05). ATO = atovaquone, MET = metformin, PHEN = phenformin.

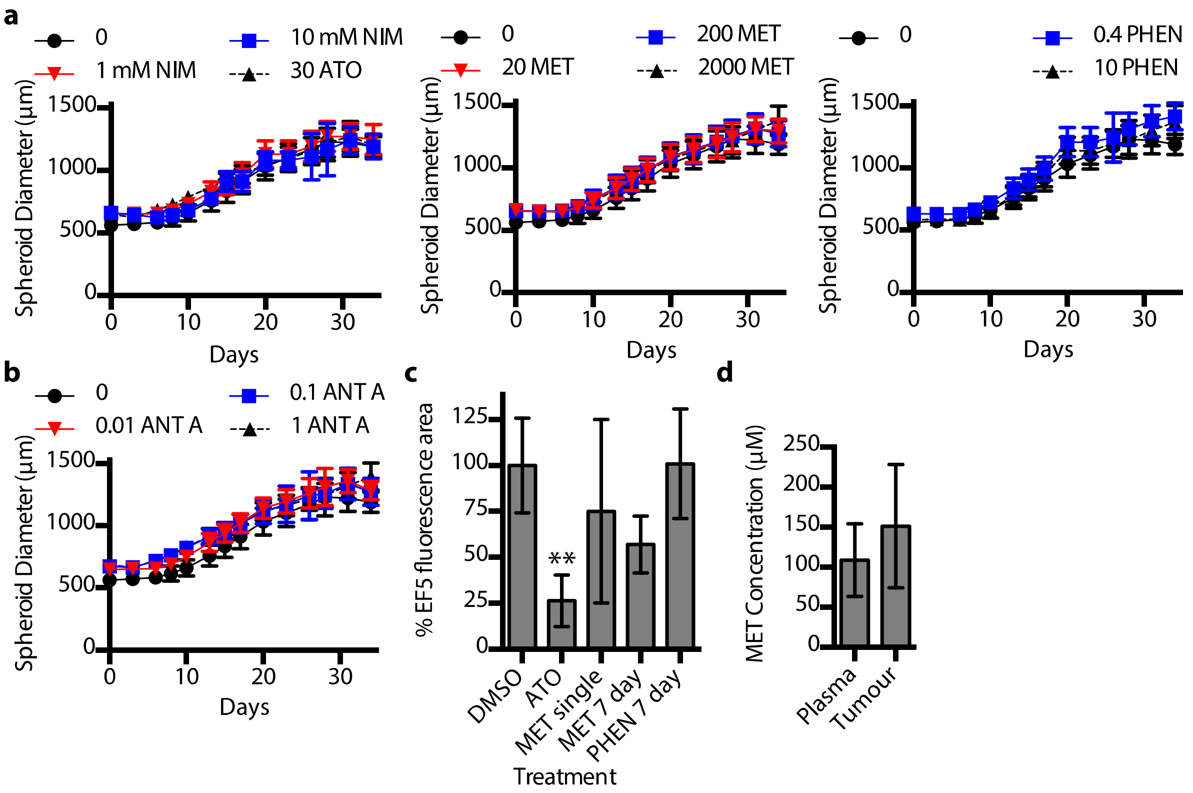

Supplementary Figure 5 | The effect of biguanides on spheroids and tumour hypoxia.

(**a** and **b**) Experiment as described in 6d and e, except that the spheroids were not irradiated, and spheroid diameter was measured for 34 days. Time post-treatment is shown (days). One replicate of the experiment was conducted with at least 27 spheroids in each treatment group. (**c**) Experiment as described in 6f. % EF5 fluorescent area is presented relative to the DMSO

treated tumours. (**d**) Mean concentration of metformin in the blood plasma and tumours after a single dose of 250 mg/kg. A one-way ANOVA was performed to assess statistical significance with Bonferroni post correction (** $P<0.01$). All values are presented as mean±SD except for the HPLC data, which are presented as mean±SEM. ATO = atovaquone, MET = metformin, PHEN = phenformin, NIM = nimorazole, ANT A = antimycin A.

		Concentratio	Exclusion criteria	Referenc
1	PYRVINIUM	10	Antihelminth with poor bioavailability	[1]
2	BERBERINE	10	Known radiosensitiser and hypoxia modifier	[2]
3	NICLOSAMIDE	10	Antihelminth with poor bioavailability	[3]
4	ACRIFLAVINIUM	10	Known radiosensitiser	[4]
5	SORAFENIB	10	Known radiosensitiser	[5]
6	EMETINE	10	Unsuitable safety profile (causes cardiac arrythmia, and gastrointestinal	[6]
7	DACTINOMYCIN	2	Previously reported plasma concentration is too low (20 nM)	[7]
8	PLICAMYCIN	10	Previously reported plasma concentration is too low (0.37 μM)	[8]
9	SULOCTIDIL	10	Unsuitable safety profile (no longer used clinically due to hepatotoxicity)	[9]
1	TENIPOSIDE	2	Unsuitable safety profile (Hypersensitivity, haematological toxicity, alopecia)	[10]
1	PENTAMIDINE	10	Previously reported plasma concentration is too low (1.03 μM)	[11]
1	DAUNORUBICIN	2	Previously reported plasma concentration is too low (37.9 nM)	[12]
1	THIOGUANINE	2	Known radiosensitiser	[13]
1	AMSACRINE	10	Unsuitable safety profile (vomiting, anaemia, haematological toxicity,	[10]
1	PHENFORMIN	10	Already studied in the context of reducing the OCR in cancer cells	[14]
1	IRINOTECAN	10	Known radiosensitiser	[15]
1	ITRACONAZOLE	10	Previously reported plasma concentration is too low (2.8 μM)	[16]
1	MITOMYCIN	10	Known radiosensitiser	[15]
1	ATOVAQUONE	10		
2	HYDROXYPROGESTERON	10	Previously reported plasma concentration is too low (56.8 nM)	[17]
2	CYCLOSPORINE	10	Shown to increase cancer risk	[18]
2	LANATOSIDE C	0.4	Metabolised in vivo to digoxin, which has a low plasma concentration of 39	[19]
2	MERCAPTOPURINE	2	Previously reported plasma concentration is too low (0.48 μM)	[20]
2	QUINACRINE	2	Unsuitable safety profile (causes psychosis, fever and vomiting)	[6]
2	FENOFIBRATE	10	Metabolised in vivo to fenofibric acid, which doesn't affect the OCR	[21]

Supplementary Table 1 | The highest ranked compounds that reduce the OCR of FaDu cells and their exclusion criteria

Cell line	O_2 (%)	0 µM	20 µM
FaDu	20	7.67	4.04
FaDu	<0.1	6.67	3.16
HCT116	20	48	40.83
HCT116	<0.1	44.92	39.33
H1299	20	23.33	13.42
H1299	<0.1	17.08	8.33

Supplementary Table 2 | Plating efficiencies at 0 Gy (%) for the colony formation assays shown in Supplementary Fig. 3c.

Supplementary References

1 Esumi, H., Lu, J., Kurashima, Y. & Hanaoka, T. Antitumor activity of pyrvinium pamoate, 6-(dimethylamino)-2-[2-(2,5-dimethyl-1-phenyl-1H-pyrrol-3-yl)ethenyl]-1-methyl-qu inolinium pamoate salt, showing preferential cytotoxicity during glucose starvation. *Cancer Sci* **95**, 685-690 (2004).

2 Peng, P. L., Kuo, W. H., Tseng, H. C. & Chou, F. P. Synergistic tumor-killing effect of radiation and berberine combined treatment in lung cancer: the contribution of autophagic cell death. *International journal of radiation oncology, biology, physics* **70**, 529-542, doi:10.1016/j.ijrobp.2007.08.034 (2008).

3 Devarakonda, B., Hill, R. A., Liebenberg, W., Brits, M. & de Villiers, M. M. Comparison of the aqueous solubilization of practically insoluble niclosamide by polyamidoamine (PAMAM) dendrimers and cyclodextrins. *Int J Pharm* **304**, 193-209, doi:10.1016/j.ijpharm.2005.07.023 (2005).

4 Lim, M. J. *et al.* Acriflavine enhances radiosensitivity of colon cancer cells through endoplasmic reticulum stress-mediated apoptosis. *The international journal of biochemistry & cell biology* **44**, 1214-1222, doi:10.1016/j.biocel.2012.04.022 (2012).

5 Plastaras, J. P. *et al.* Cell cycle dependent and schedule-dependent antitumor effects of sorafenib combined with radiation. *Cancer Res* **67**, 9443-9454, doi:10.1158/0008-5472.CAN-07-1473 (2007).

6 Khaw, M. & Panosian, C. B. Human antiprotozoal therapy: past, present, and future. *Clin Microbiol Rev* **8**, 427-439 (1995).

7 Veal, G. J. *et al.* Pharmacokinetics of dactinomycin in a pediatric patient population: a United Kingdom Children's Cancer Study Group Study. *Clin Cancer Res* **11**, 5893-5899, doi:10.1158/1078-0432.CCR-04-2546 (2005).

8 Fang, K. *et al.* Determination of plicamycin in plasma by radioimmunoassay. *Ther Drug Monit* **14**, 255-260 (1992).

9 Chung, M. W., Komorowski, R. A. & Varma, R. R. Suloctidil-induced hepatotoxicity. *Gastroenterology* **95**, 490-491 (1988).

10 Pai, V. B. & Nahata, M. C. Cardiotoxicity of chemotherapeutic agents: incidence, treatment and prevention. *Drug Saf* **22**, 263-302 (2000).

11 Bronner, U. *et al.* Pentamidine concentrations in plasma, whole blood and cerebrospinal fluid during treatment of Trypanosoma gambiense infection in Cote d'Ivoire. *Trans R Soc Trop Med Hyg* **85**, 608-611 (1991).

12 Bellott, R. *et al.* Pharmacokinetics of liposomal daunorubicin (DaunoXome) during a phase I-II study in children with relapsed acute lymphoblastic leukaemia. *Cancer Chemother Pharmacol* **47**, 15-21, doi:10.1007/s002800000206 (2001).

13 Kim, J. H., Alfieri, A. A., Kim, S. H. & Hong, S. S. Radiosensitization of two murine fibrosarcomas with 6-thioguanine. *International journal of radiation oncology, biology, physics* **18**, 583-586 (1990).

14 Wheaton, W. W. *et al.* Metformin inhibits mitochondrial complex I of cancer cells to reduce tumorigenesis. *eLife* **3**, e02242, doi:10.7554/eLife.02242 (2014).

15 Candelaria, M., Garcia-Arias, A., Cetina, L. & Duenas-Gonzalez, A. Radiosensitizers in cervical cancer. Cisplatin and beyond. *Radiation oncology* **1**, 15, doi:10.1186/1748-717X-1-15 (2006).

16 Hardin, T. C. *et al.* Pharmacokinetics of itraconazole following oral administration to normal volunteers. *Antimicrobial agents and chemotherapy* **32**, 1310-1313 (1988).

17 Caritis, S. N. *et al.* Pharmacology and placental transport of 17-hydroxyprogesterone caproate in singleton gestation. *Am J Obstet Gynecol* **207**, 398 e391-398, doi:10.1016/j.ajog.2012.08.015 (2012).

18 Hojo, M. *et al.* Cyclosporine induces cancer progression by a cell-autonomous mechanism. *Nature* **397**, 530-534, doi:10.1038/17401 (1999).

19 Smith, T. W. Pharmacokinetics, bioavailability and serum levels of cardiac glycosides. *J Am Coll Cardiol* **5**, 43A-50A (1985).

20 Innocenti, F. *et al.* Clinical and experimental pharmacokinetic interaction between 6-mercaptopurine and methotrexate. *Cancer Chemother Pharmacol* **37**, 409-414, doi:10.1007/s002800050405 (1996).

21 Wilk, A. *et al.* Molecular mechanisms of fenofibrate-induced metabolic catastrophe and glioblastoma cell death. *Molecular and cellular biology* **35**, 182-198, doi:10.1128/MCB.00562-14 (2015).
